# Genetic Manipulation of Glycogen Allocation Affects Replicative Lifespan in *E*. *coli*

**DOI:** 10.1371/journal.pgen.1005974

**Published:** 2016-04-19

**Authors:** Alex Boehm, Markus Arnoldini, Tobias Bergmiller, Thomas Röösli, Colette Bigosch, Martin Ackermann

**Affiliations:** 1 Biozentrum, University of Basel, Switzerland; 2 Philipps-Universität Marburg, LOEWE-Zentrum für Synthetische Mikrobiologie, Marburg, Germany; 3 Institute of Biogeochemistry and Pollutant Dynamics, Department of Environmental Systems Science, ETH Zurich, Switzerland; 4 Department of Environmental Microbiology, Eawag, Dübendorf, Switzerland; 5 Department of Physics, University of California, San Diego, La Jolla, United States of America; 6 Institute of Science and Technology Austria, Klosterneuburg, Austria; Universidad de Sevilla, SPAIN

## Abstract

In bacteria, replicative aging manifests as a difference in growth or survival between the two cells emerging from division. One cell can be regarded as an aging mother with a decreased potential for future survival and division, the other as a rejuvenated daughter. Here, we aimed at investigating some of the processes involved in aging in the bacterium *Escherichia coli*, where the two types of cells can be distinguished by the age of their cell poles. We found that certain changes in the regulation of the carbohydrate metabolism can affect aging. A mutation in the carbon storage regulator gene, *csrA*, leads to a dramatically shorter replicative lifespan; *csrA* mutants stop dividing once their pole exceeds an age of about five divisions. These old-pole cells accumulate glycogen at their old cell poles; after their last division, they do not contain a chromosome, presumably because of spatial exclusion by the glycogen aggregates. The new-pole daughters produced by these aging mothers are born young; they only express the deleterious phenotype once their pole is old. These results demonstrate how manipulations of nutrient allocation can lead to the exclusion of the chromosome and limit replicative lifespan in *E*. *coli*, and illustrate how mutations can have phenotypic effects that are specific for cells with old poles. This raises the question how bacteria can avoid the accumulation of such mutations in their genomes over evolutionary times, and how they can achieve the long replicative lifespans that have recently been reported.

## Author Summary

Bacteria were often considered to be potentially immortal and free of aging. This expectation was based on the idea that the two cells emerging from bacterial division are identical and thus also equally old. However, a number of recent studies followed individual bacterial cells over consecutive divisions and reported that individuals with old cell poles show reduced survival and growth. This indicates that at least some types of bacteria age. We were interested in how mutations can affect the aging process and replicative lifespan of bacteria. In eukaryotes the aging process is thought to be modulated by mutations with age-specific effects. For example, there are mutations that have no measureable phenotypic effect early in life but are deleterious later in life. Such mutations play a key role in the evolution of eukaryotic aging and are thought to be responsible for lifespan differences between species. Here, we report that mutations can also have age-specific effects in bacteria. We describe a mutation in *E*. *coli* with a deleterious effect on growth and division that only manifests in cells whose cell pole is about four to five divisions old. These results illustrate how mutations can act in an age-specific manner in bacteria, and raise questions about how bacterial lifespan is modulated by such mutations.

## Introduction

Long-term observation of individual cells of different species of rod-shaped bacteria showed that there is an association between the age of a cell’s poles and the performance of this cell [1–6]. If one focuses on a cell pole from the moment it is formed during cell division, and follows it over successive divisions, one can observe that the rate of reproduction or survival of the cell carrying this pole declines, and that the cell thus ages [1–6]. Cell division is therefore asymmetric with respect to the potential for division and survival: the cell that receives the older pole can be regarded as an aging mother, and the cell receiving the younger pole as a rejuvenated daughter.

The observation of aging in bacteria is consistent with a basic insight from the evolutionary theory of aging: that aging exists because selection against it is weak [7,8]. In nature, most organisms die for external reasons, usually at a much earlier age than they could maximally achieve. As a consequence, mutations that have deleterious effects that manifest only late in life are not efficiently selected against, because most of the carriers of such mutations will have reproduced and died before the deleterious effects manifest. Such mutations can thus accumulate in the genome over evolutionary time scales and contribute to aging. This argument can also be adopted for bacteria: very few individuals in a bacterial population have a cell pole with a large divisional age (i.e., a cell pole that was formed many divisions ago), and bacterial populations are dominated by cells with young poles. Following from this argument, mutations with a deleterious effect that is specific for cells with old poles are only weakly selected against, and could cause or contribute to bacterial aging.

**Fig 1:**
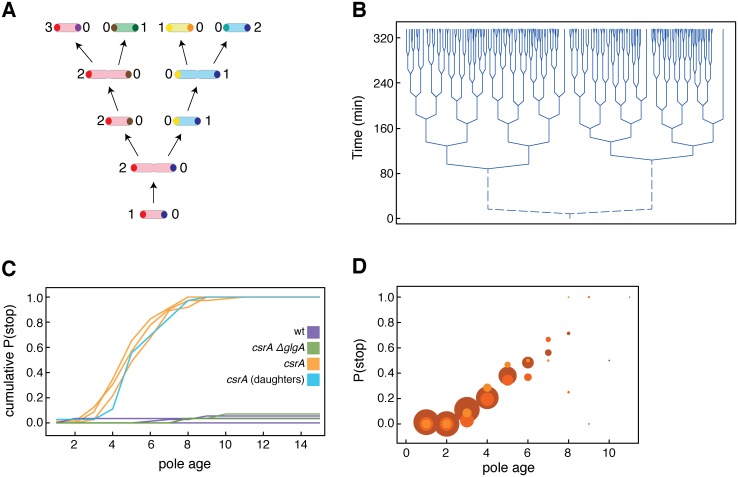
*CsrA* mutant cells stop dividing at low pole ages. (**A**) illustrates the concept of pole age. Every cell has one pole of age zero that has been formed at the last division (the young pole), and one pole of age one or more (the older pole). We use the older pole to define individuals, and the age of the older pole to assign a pole age to each individual. A new individual emerges from division as a cell with an older pole of age 1. If this cell divides, its older pole segregates to one of the two cells emerging from division, and increases its age to two. We refer to the cell receiving this pole as the same individual that underwent division (since it has the same older pole). This individual can be referred to as the ‘mother’ of the other cell that emerges from division. This other cell—the ‘daughter’–has an older pole of age one. We refer to this other cell as a new individual. In panel A, we use colors to mark individuals. The first individual is light red, and its older pole is dark red. This individual produces two daughters, the blue individual and the green individual. (**B**) shows a representative lineage tree of a microcolony of *csrA* mutant cells. Each branching event in the lineage tree corresponds to one cell division. The lineage tree is organized according to pole age. At each cell division, the branch that represents the daughter—the cell with the new cell pole—extends to the left; the branch that represents the mother—the cell with the older pole—extends to the right. Three individuals with old poles stop dividing. Note that these experiments are initiated with single cells whose old and new pole we cannot distinguish; the branches representing the first cell division are thus dashed. In (**C**), we show that the cumulative probability of *csrA* mutant cells to stop dividing increases with increasing pole age (orange lines; see Materials and Methods for how we defined when a cell stops dividing; the median pole age at which *csrA* mutant cells stop dividing is 4). For wild type (purple) and *csrA ΔglgA* double mutant cells (green) very few cells were observed to stop dividing. Three independent experiments were performed for *csrA* mutant cells (N = 38, 71, 23), and two independent replicates were performed for the other two strains (N = 25, 74 for wild type, N = 31, 28 for *csrA ΔglgA*). (**D**) shows that the *per division* probability of *csrA* mutant cells to stop dividing increases with increasing pole age, for the same three experiments (denoted by different shades of orange). The diameter of the circles is proportional to the sample size at each pole age, i.e., to the number of cells that were still alive. In all three experiments, the probability to stop increases significantly with increasing pole age (logistic regression, p<0.001 for all three experiments, N = 38, 71, 23).

While the existence of bacterial aging is thus in line with the evolutionary theory of aging, there is a puzzling quantitative discrepancy between expectation and observation: bacteria live much longer than a simple theoretical consideration would suggest. Two studies—one with *Caulobacter crescentus* [1], the other with *E*. *coli* [6]–followed single cells for long times, and reported that individual bacterial cells can survive and divide for more than one hundred divisions. The observation with *E*. *coli* is particularly surprising, as can be shown with a simple argument: in bacterial populations where cells with different pole ages are exposed to the same external conditions, the distribution of pole ages is exponential: 50% of the cells have a pole of age one, 25% have a pole of age two, and so on (for a definition of the pole age of a bacterial cell see Fig 1A). More formally, the fraction of cells with a pole age *n* is 2-*n*; i.e. only about one in a million cells has a pole age of 20 or more. Therefore, there is virtually no selection for cells to remain viable once their cell pole is 40 or 50 divisions old (this argument assumes that the process of aging in this case depends on replicative rather than chronological age). Importantly, the pole age distribution in a bacterial population in which all cells are exposed to the same external conditions and extrinsic mortality is stable and not dependent on the growth dynamics of the population [9,10].

Why do bacterial cells then continue surviving and dividing and reach such high reproductive ages? The argument outlined above suggests that selection alone cannot account for this. One possible explanation is thus that mutations with negative effects that affect exclusively old-pole cells simply do not exist. It is conceivable that all mutations that have a detrimental effect in cells with old cell poles always also have some (possibly much smaller) detrimental consequences that manifest in cells with younger poles, so that selection would remove these mutations from bacterial populations over evolutionary times.

Here, we report the fortuitous discovery of a mutation with an age-specific effect on growth and division in *E*. *coli*, a discovery that challenges the hypothesis that mutations acting specifically on old-pole cells do not exist. The discovery came out of a study aimed at identifying genetic changes that influence the replicative lifespan of individual bacterial cells. From eukaryotes as diverse as yeast and mammals, it is known that the consumption and allocation of carbohydrates and other nutrients has profound effects on aging [11–13]. We wanted to find out whether similar effects play a role in bacteria, and therefore introduced a genetic change to modify the allocation of carbohydrates inside the bacterial cell. In *E*. *coli*, one important regulator for the processing of carbohydrates is the carbon storage regulator gene *csrA* [14]. Certain mutations in this locus lead to metabolic changes [15] that are superficially similar to processes in aging eukaryotic cells [16,17]. We thus tested whether the changes brought about by a mutation in *csrA* would decrease replicative lifespan in *E*. *coli*.

To assay aging and replicative lifespan, we used single-cell observation of bacteria growing on a solid substrate and in microfluidic devices. In such experiments, individual bacteria are kept under conditions where they divide and give rise to clonal families. These families can be observed under the microscope, and cell division events can be monitored. One can then analyze whether division and survival rates of individual bacteria change with age. We define the age of an individual as the age of its cell pole, or, more precisely, as the age of its *older* cell pole (Fig 1A). Every cell in rod shaped bacteria has two poles, one of which was formed at the last division. The other pole is older, and we use it here to define the age of the cell, in agreement with previous studies [2,4,5,18]. An individual is born at age one, when the pole defining it becomes the older cell pole (Fig 1A). By following this pole, one can follow an individual over successive divisions. Aging manifests as a decrease in the growth or survival of this individual with increasing age [1–6]. Using this approach, we found that cells with the *csrA* mutation stop dividing once their old pole is about five divisions old, and investigated the cellular processes that lead to the termination of division.

While our findings are most likely—in contrast to our initial expectations—not related to dietary restriction in eukaryotes, they provide a new perspective on fundamental aspects of aging in bacteria, and possibly of cellular aging in general. Bacteria were for a long time considered to be free of aging and potentially immortal, because it was assumed that deleterious mutations would affect both individuals emerging from reproduction equally (and thus not be age-specific in their effect, [19]). In contrast to this viewpoint, the results reported here show that mutations can indeed have age-specific effects, and provide first insights into the cellular mechanisms that can lead to age-specificity. While mutations that reduce lifespan might often not be directly related to the normal aging process [20], we think that studying the effects of such mutations in bacteria can help understand fundamental principles of bacterial aging, and how an old and a young bacterial cell can arise from the same cell division.

## Results/Discussion

We introduced a mutant allele (*csrA*::(T*n5*Δ*kan*::*Frt)*;[14]) into the carbon storage regulator locus *csrA*. This mutation has the following consequences: it leads to increased glycogen production, inhibits glycolysis, and increases gluconeogenesis [15]. The transposon insertion mutation confers a partial loss of function; *csrA* is essential under the conditions used here [21]. Microscopic observation of individual cells showed that the mutation in *csrA* had a strong effect on replicative lifespan (Figs 1B and S1, S1 and S2 Movies). We followed cells from the moment they emerged as new-pole individuals, and analyzed growth and division as a function of the age of their cell pole. In the cells with the *csrA* mutation, the probability that a cell will stop growing and dividing increased markedly within the first ten divisions, and the pole age where 50% of the population had stopped dividing was estimated to be 3.76 (Fig 1C).

A more detailed analysis revealed that this decline was not a consequence of a constant mortality rate; rather, the probability that a cell would stop to divide after a given division increased markedly with increasing pole age. Cells almost never stopped dividing when their older pole was one or two divisions old; when their older pole increased in age, the probability to stop dividing also increased and reached over 50% for cells whose pole was six divisions old or older (Fig 1D). Such a reduction in the rate of reproduction and survival with age is consistent with the definition of aging [22]. This observation thus indicates that the mutation in *csrA* leads to faster aging, and that the short reproductive lifespan is a consequence of this faster aging.

To further analyze cellular changes that accompanied cessation of division, we measured single-cell elongation rates of cells that stopped to divide. Cells still elongated after the last division, but the length increase was very small (mean length changes in the 25 minutes following the last division compared to cell length at last division, based on three independent experiments: 7.4%, 7.2%, 7.6%, standard deviations: 5.6%, 7.4%, 5.8%, N: 37, 72, 23). We found that the length of a cell after cell division was predictive of its remaining replicative potential: cells that did not divide again emerged at a smaller size from division (S2 Fig).

Importantly, the growth and division-terminating phenotype of the *csrA* mutation affected two cells emerging from division differentially. This is most clearly seen at division events where one cell—the old-pole mother—stops dividing (for example the division that gives rise to the right-most branch in the lineage tree in Fig 1B). The other cell emerging from division is a new individual with a new pole. The time it takes this new individual to initiate the first division as well as the total replicative potential (the number of divisions that an individual can make before it stops) are typical for new-pole cells: First, we analyzed the time it took every individual (defined based on a given cell pole, as shown in Fig 1A) to complete its first division, and compared it to the time it took the individual’s last daughter to accomplish its first division. The difference between the two groups was not statistically significant (Anova, p = 0.37; S3 Fig). Second, by comparing the orange curves (*csrA* mother cells) with the blue curve (last daughters of *csrA* mother cells) in Fig 1C, it becomes evident that the total replicative potential that each individual had at the moment it emerged as a young pole cell is indistinguishable from the total replicative potential of its last daughter.

This means that the negative phenotypic effect of the *csrA* mutation acts in an age-specific manner; cells that carry this mutation are only affected once their cell pole has reached a high age. When these cells divide, they give rise to new-pole daughters that reset their biological clock with respect to the onset of the growth and division-terminating phenotype, and these daughters continue to divide when the old pole mother cell has long stopped. While this effect is unusual for bacteria, it corresponds to how genes are thought to affect aging in eukaryotes. Indeed, mutations that contribute to aging can only escape the purging effect of natural selection if their negative effects manifest late in life, and if the progeny of these aging individuals are born rejuvenated [3,7,9,23].

As discussed in the introduction, such mutations with a negative effect that acts specifically on old individuals are not strongly selected against. Because the negative effect of the *csrA* mutation only affects cells whose pole is about four or more divisions old, and the progeny of these cells are rejuvenated, only a relatively small proportion of the cells in a population should be affected by the division-terminating phenotype at any point in time. As a consequence, populations carrying this mutation are expected to grow exponentially, and at a rate not much lower than the wild type. To address this issue more quantitatively, we constructed a simple mathematical model based on the Euler-Lotka equation to estimate the reduction in population growth rate resulting from the age-dependent mortality depicted in Fig 1C and 1D. This model showed that a strain with this mortality pattern has a population growth rate that is only about 6% lower than a strain with the low age-specific mortality observed in the wild type (S1 Text).

We then compared this theoretical prediction to experimental growth rate data. We initiated 48 populations of the *csrA* mutant and 48 populations of an isogenic strain with the wild type *csrA* allele, and analyzed their growth dynamics. Both types of populations grew exponentially (S4 Fig), and at similar rates: the fast aging *csrA* strain had a doubling time of 42.9±1 min (mean±SE), and the strain with the wildtype *csrA* allele had a doubling time of 42.6±0.8 min (mean±SE). The growth rates of the two strains were not significantly different (p = 0.67; 95% confidence interval for the difference between the doubling time of the *csrA* and the wild type: -1.2 minutes / +1.9 minutes). This means that the empirically determined growth deficit of the *csrA* strain was significantly smaller than predicted by the theoretical model. A possible reason for this discrepancy is the difference in the growth conditions: the theoretical model was parametrized based on measurements in the microfluidic device, while the empirically measured growth curve was based on populations growing in a batch culture. Alternatively, it is also possible that the *csrA* batch cultures became dominated by phenotypic or genetic variants that restore longer replicative lifespan, or that the old pole cells that stop dividing also were enriched for detrimental cellular components and thereby purged these components from the growing populations.

However, and importantly, both the model and the experiments emphasize the point that deleterious mutations that manifest only in cells with older poles only lead to a moderate reduction in a genotype’s long term growth rate. In the case of the *csrA* mutant the predicted reduction (6%, or 0.06) is so large that it would be opposed by natural selection. In general, selection is effective in removing deleterious mutations whose detrimental effect is in the order of the reciprocal of the effective population size [24] or more. The effective population size of *E*. *coli* (relevant for selection) is about 105 [25]. This means that only mutations that lead to a reduction of the long-term growth rate by about 10–5 or less can escape purifying selection (i.e., the effect on the long term growth rate would need to be about 0.00001 instead of the 0.06 predicted for the *csrA* mutant). If the deleterious effect if the *csrA* mutation would manifest at a higher replicative age, when cells have a pole age of around 17 to 18 (instead of four to five as reported in Fig 1), then the resulting reduction of the populations long-term growth rate would be less than 0.00001 (S1 Text), and natural selection would not be effective in removing such mutations from bacterial populations. The *csrA* mutation thus established that mutations with age-specific deleterious effects can arise in bacteria, and can serve to illustrate how mutations whose deleterious phenotypic effect only affects cells with old poles are not efficiently removed by natural selection.

Next, we aimed at understanding the cellular and molecular processes that lead to the termination of division in the *csrA* mutant. It is well documented that the *csrA* mutation leads to an accumulation of glycogen in the cells [14]. We thus asked whether accumulation of intracellular glycogen could be involved in the short replicative lifespan of the *csrA* mutant. To investigate this question, we tested whether the disruption of glycogen synthesis could rescue the short replicative lifespan. We deleted the gene *glgA* in the *csrA*::*Tn5* background. *glgA* encodes glycogen synthase and is essential for glycogen biosynthesis [26]. Intriguingly, this manipulation completely rescued the life-shortening effect of the *csrA* mutation: cells carrying both mutations were indistinguishable from wild type *E*. *coli* in the rate at which their reproductive ability changed with age (Fig 1C).

This suggested that the accumulation of glycogen in the *csrA* mutant has a direct or indirect negative effect on replicative lifespan. That this negative effect only affects the old-pole cells could be a consequence of segregation of glycogen to these cells. Macromolecules can segregate to old-pole cells [4,27] through diffusion and spatial exclusion with the nucleoid [28]. Previous electron microscopy experiments showed that glycogen localizes to the cell poles in *E*. *coli* [29] and *Salmonella* [30]; polar localization would offer a mechanism for asymmetric distribution of glycogen and thereby the phenotypic difference between the old-pole and the new-pole cell.

**Fig 2:**
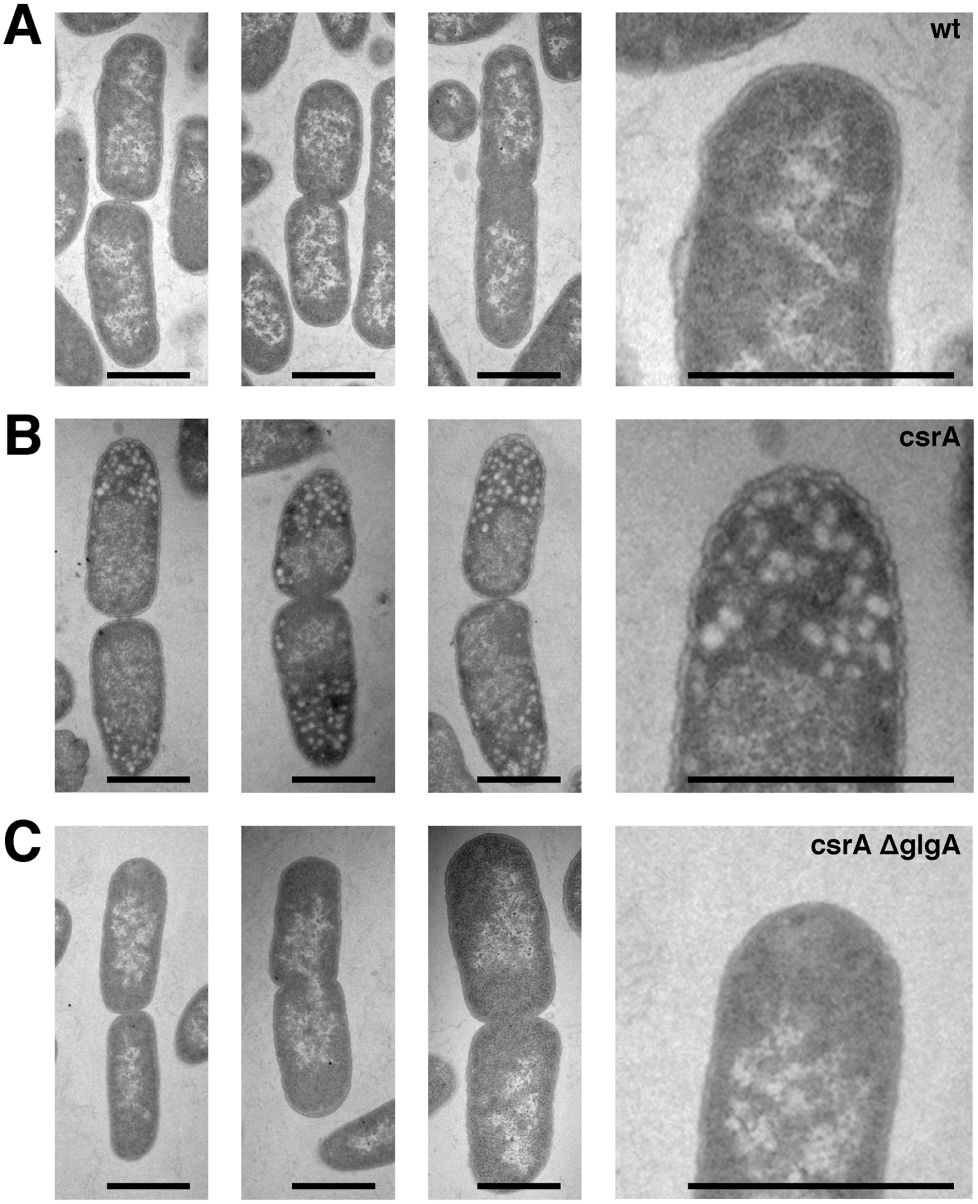
Electron micrographs show polar localization of glycogen in csrA mutant cells. Electron micrographs of wild type *E*. *coli* cells (**A**), *csrA* mutant cells (**B**) and *csrA ΔglgA* double mutant cells (**C**). Three representative pre-divisional cells per strain are depicted. The last image in each row is a magnified view of the first image in this row. Only the *csrA* mutant (**B**) displays granular structures indicative of glycogen, and these structures localize to the cell poles (in these experiments, it is not possible to determine which of the two cell poles is the old pole). Cells from the other two strains show no evidence of glycogen granules. Size bars are 1μm.

We used two approaches to investigate whether and how glycogen localizes in the *E*. *coli* strains that we had investigated for their aging phenotype. First, we analyzed cells with electron microscopy, and found that, in the *csrA* mutant strain, granules that have been interpreted as glycogen [29,30] localized almost exclusively at the cell poles (Fig 2B); *E*. *coli* wild type cells and the *csrA ΔglgA* double mutant did not show evidence for the accumulation of glycogen granules (Fig 2A and 2C). If newly formed poles are initially free of glycogen, and if glycogen continuously accumulates with increasing pole age, this would directly lead to a difference in the amount of cellular glycogen between cells with old and young poles, and thus could explain their different fates if glycogen has a negative effect on the ability to grow and divide. However, in these experiments, we lack information on the pole age of the cells observed, and we are thus not able to determine whether glycogen accumulates specifically at the *old* cell poles.

**Fig 3:**
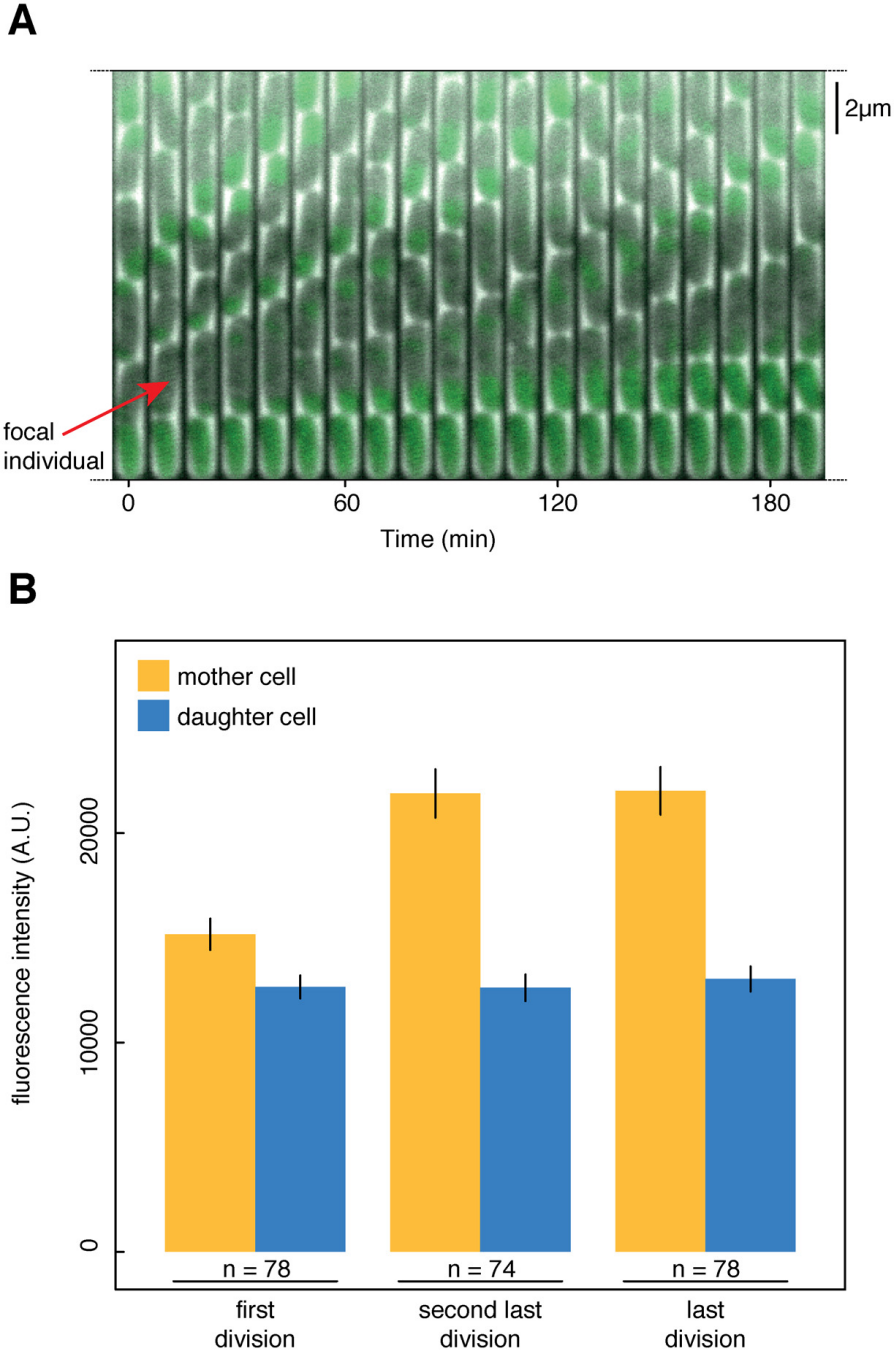
GlgA-GFP indicates polar localization of glycogen in *csrA* mutant cells. *CsrA* mutant cells harboring a plasmid encoding GlgA-GFP fusion protein were observed by time-lapse microscopy when growing in microfluidic devices. (**A**) shows a temporal montage of still images from different time points, starting with the emergence of a new individual (‘focal individual’) at time 10 min that produces three daughters and then stops dividing. GlgA-GFP signal accumulates at the old pole and eventually fills the whole cell. The focal individual is the last daughter of the cell at the bottom of the channel. (**B**) is a quantification of fluorescence intensity of the focal individual (‘mother cell’, yellow) and its young pole daughter cells (blue), for the focal individual’s first, second last, and last division. Fluorescence is extracted as integrated density. GlgA-GFP signal stays approximately the same in all daughter cells, but accumulates in mother cells with increasing pole age. Error bars denote standard error of the mean.

We thus used a second approach where we analyzed the intracellular localization of a fusion protein of the glycogen synthase GlgA to the green fluorescent protein (GFP). It has been reported that GlgA cocrystallizes with oligosaccharides [31], indicating that determining the localization of GlgA can inform us about the spatial distribution of glycogen in the cell. The GlgA-GFP fusion protein was preferentially localizing at the old poles of *csrA* mutant cells (Fig 3A, S3 Movie), and also, yet to a lesser extent, of wild type cells (S4 Movie). More importantly, the GlgA-GFP signal accumulates at old poles of *csrA* mutant cells over consecutive divisions (Fig 3A), and after the last division event, GlgA-GFP appeared to be distributed across the whole cytoplasm (Fig 3A). We quantified the distribution of GlgA-GFP for mother and daughter cells of first, second last, and last divisions in *csrA* mutant cells (Fig 3B). The results support a gradual accumulation of glycogen at the old pole that eventually leads to cells that are filled with glycogen (S5 Fig). Analysis of the GlgA-GFP concentration inside a cell and the cell’s length after division showed that both traits are predictive of whether the observed division is this cell’s final division or not (S6 Fig). Additionally, a cell’s GlgA-GFP concentration and cell size when it emerges as a new-pole cell is predictive of its total replicative potential; the brighter and smaller cells are ‘at birth’ when they emerge as a new-pole cell, the lower the total replicative lifespan they will reach (S7 Fig). As a control, we observed cells expressing GFP alone, without fusion to another protein, under control of the promoter for the ribosomal protein RpsM; we found no evidence for the distribution of GPF being different between young and old pole daughter cells, showing that the effect observed with GlgA- GFP is not due to passive accumulation of GFP molecules at the cell poles (S8 Fig).

**Fig 4:**
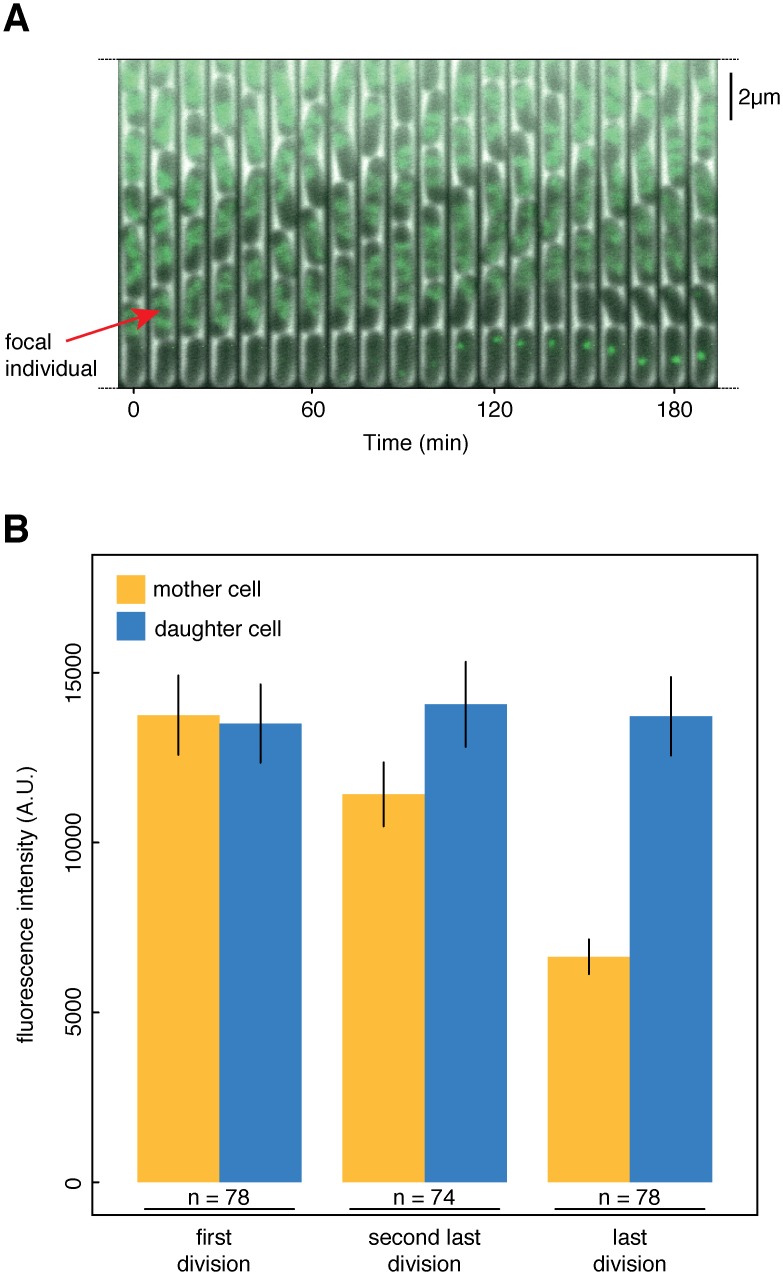
Hns-GFP is excluded from old pole cells in the *csrA* mutant. *csrA* mutant cells harboring a plasmid encoding Hns-GFP were grown in microfluidic devices and observed by time-lapse microscopy. (**A**) shows a temporal montage of still images analogous to Fig 3A, showing the entire lifetime of a focal individual. (**B**) is a quantification of fluorescence intensity of the focal individual (‘ mother cell’, yellow) and its young pole daughter cells (blue), analogous to Fig 3B. Hns-GFP reaches similar levels in daughter cells regardless of the mother’s age, but drops dramatically at a cells last division, indicating loss of the mother cell’s chromosome. Error bars denote standard error of the mean.

How could the accumulation of glycogen at the old cell pole lead to the termination of growth and division of the cell carrying this pole? Previous studies reported mutual spatial exclusion between (protein) aggregates and the bacterial chromosome [27, 28], raising the possibility that the large polar aggregates in the *csrA* mutant could lead to the exclusion of the chromosome. To test whether spatial exclusion of the chromosome by the observed glycogen accumulation leads to the short replicative lifespan in this strain, we followed the localization of a fusion protein of Hns to GFP. Hns is a protein that binds to AT-rich regions on DNA [32], and can thus be used as a marker for the location of the chromosome if fused to GFP. Hns-GFP was excluded from older pole cells in the *csrA* mutant, and this exclusion became apparent already at early pole ages (Fig 4A, S5 Movie). After the last division, no visible Hns-GFP signal remained in the old pole daughter cell, indicating a loss of chromosomal DNA (Fig 4A and 4B). We measured Hns-GFP intensity in mother and daughter cells of first, second last, and last divisions (Fig 4B), and found a drop in fluorescence in mother cells at the last division, indicating a loss of the chromosome. We scrutinized this finding using a DNA stain; again, after the last division, the old pole daughter cells did not contain visibly stained DNA (S9 Fig). These results thus indicate that the glycogen accumulating at the old pole in cells of the *csrA* mutant excludes the chromosome, which would explain why these cells then stop growing and dividing. Bacterial cells without chromosomes have been observed before, most prominently in the case of ‘minicells’ [33]; these minicells are produced as a consequence of mutations in cell division genes [34], they are about 10 times smaller than normal cells [33], and there is (as far as we know) no evidence that the loss of the chromosome is specific for cells with old cell poles. In contrast, the *csrA* cells lose their chromosome ultimately as a consequence of a metabolic change, these cells are only slightly smaller than other cells in the population (S2 Fig), and the loss of the chromosome is specific for cells with old cell poles. While these two observations share the aspect of chromosome deficiency, they are thus different in a number of other aspects.

Our results provide new insights in principles of aging and rejuvenation at the level of individual cells. At this level, a distinction between an aging mother and a rejuvenated daughter requires that waste products and aged subcellular structures are not distributed randomly, but preferentially segregate to one cell during division. In eukaryotes, we are starting to gain a molecular understanding of how two cells emerging from division can differ in their age and thus in performance [35–37]. The results presented here show that changes in the accumulation of cellular storage compounds can affect the two bacterial cells emerging from division differentially, thus contributing to a difference in their fate. As in a previous study [4], we find evidence that the localization of macromolecules to specific positions in the cell might promote the asymmetric distribution of these molecules. In the case of the *csrA* mutant, the accumulation of these macromolecules was associated with the loss of the chromosome, possibly because of spatial exclusion between these two components. This mechanism also provides a plausible explanation for the rejuvenating effect reported in S3 Fig: the loss of growth and division associated with old pole age only arises in cells in which the glycogen level at the cell pole is high enough to exclude the chromosome.

It is important to note that the details of the cellular mechanism underlying short replicative lifespan in the *csrA* mutant do probably not extend beyond bacteria. Spatial exclusion between glycogen and DNA is not a plausible molecular mechanism for eukaryotic aging, and long-lived mutants of *Drosophila melanogaster* [38] and *Caenorhabditis elegans* [39] have *higher* levels of glycogen than wild type animals, indicating that increased levels of glycogen are not generally associated with shorter life-span. However, it will be interesting to find out whether other aspects of our observations are more general: whether exclusion of the nucleoid by accumulating macromolecules is involved in aging of wildtype bacteria and possibly mitochondria, and whether spatial exclusion of other essential cellular components by accumulating macromolecules plays an important role in cellular aging and rejuvenation in eukaryotes, for example in stem cells and in the germ line.

The probably most surprising aspect of our results is the identification of a mutation with a negative effect that is specific to cells with old poles. As discussed in the Introduction, the observation of high replicative lifespans in bacteria (compared to the theoretical considerations developed in the introduction) could be explained if such mutations were not possible. Our results show that mutations with such phenotypic effects *are* possible, even if the mutation we identified affects the cells at a pole age low enough to be visible on a population level and selected against. Based on the observations with the *csrA* mutation, one might expect that there would be other mutations with similar effects that manifest at higher pole ages. Such mutations could escape the purging effect of selection, accumulate in genomes over evolutionary times, and reduce the replicative lifespan of these bacteria. Why this does not happen is currently not clear, and is in our opinion an open and fascinating fundamental question about aging in these simple organisms.

## Materials and Methods

### Bacterial strains and growth conditions

MG1665 is an Escherichia coli K-12 wildtype strain with known genome sequence [40]. The *csrA*::*Tn5 strain* AB958 is a derivative of MG1655 that was constructed as follows: The *csrA*::*Tn5*(kan) insertion from Tr1-5 [14] was P1 transduced into MG1655 following standard protocols[41]. Subsequently, the kanamycin cassette in this strain was exchanged for a chloramphenicol cassette with the help of λRED mediated allelic exchange according to [42] by employing the following oligonucleotide primers (aph_KO_P1: gagccatattcaacgggaaacgtcttgctcgaggccgcgattaaTGTAGGCTGGAGCTGCTTCG and aph_KO_P2: aaaactcatcgagcatcaaatgaaactgcaatttattcatatcaCATATGAATATCCTCCTTAG). In a last step the chloramphenicol cassette was removed by Flp recombinase mediated site-specific recombination [42]. The *csrA*::*Tn5 ΔglgA* strain AB1064 has been obtained by P1 transducing AB958 with a *ΔglgA*::*kan* allele from the “Keio” collection [43] and subsequent removal of the kanamycin cassette by Flp recombinase mediated site-specific recombination [42]. To monitor localization of translational protein-GFP fusions, MG1655 and AB958 were transformed with IPTG inducible plasmids harboring the respective constructs[44]. Strains harboring these plasmids were grown on LB medium containing 15μg/ml chloramphenicol (Sigma). Expression of the fusion proteins was induced by adding 1μM IPTG (isopropyl thiogalactopyranoside, Sigma) 30min before microscopic observation. Distribution of the GFP protein was performed using strains harboring a plasmid encoding GFP controlled by the promoter for the ribosomal protein RpsM[45]. Strains harboring this plasmid were grown on LB medium containing 50μg/ml kanamycin (Sigma). All experiments were carried out in LB liquid medium (10g/L Bacto tryptone, 5 g/L yeast extract, 10g/L NaCl) or on LB agar plates (15g/L agar) at 30°C.

### Microfluidics

Microfluidic devices were fabricated and used as described in [46].

### Light microscopy

For the colony growth assays on LB agar pads, 0.5 μl of exponentially growing bacterial culture was placed on an LB agar pad in a microscope cavity slide. The slide cavities were sealed with laboratory grease and covered with a cover slip. Time-lapse microscopy was performed as described in [46], with incubation at 30°C during the whole experiment.

### Image analysis

Time-lapse images of growing colonies were analyzed using a modified version of the Schnitzcells software developed in the Elowitz group [47]. Movies of cells growing in microfluidics devices were analyzed using the plugin MMJ [46] for ImageJ. Both analysis tools allow extracting of fluorescence intensities and cell divisions during the course of the whole experiment.

### Determination of replicative lifespan in microscopy movies

At the start of microscopy experiments, it is impossible to tell the age of the cell poles. In experiments using microfluidic devices, we therefore discarded the bottom cell in all channels in experiments with the *csrA* mutant, and analyzed the last daughter of this cell. In the experiments with wild type and the *csrA ΔglgA* double mutant, we used bottom cells, since these cells do not stop dividing during the course of the experiments. This is a conservative difference: if the bottom cells in the channels have different ages, they can only be older. A cell is considered to have stopped dividing if it does not divide any more until the end of the experiment, and if the interval between the last division of the cell and the end of the experiment exceeds the average interdivision interval by a factor of 2. We only considered cells that we could observe for at least 850min (170 frames at 5min/frame). These criteria were used to make sure that cells have indeed stopped dividing, rather than just dividing slowly, and that we allow the time to observe enough divisions.

### Measurement of GlgA-GFP concentration

To get a measure of cellular concentrations of GlgA-GFP, we used the mean GFP intensity measured in the cells, and calculated a concentration under the assumption that the cellular volume can be approximated by a cylinder. We assumed the cell length to be the height of the cylinder, and the width of the microfluidic channels (1.2μm) to be the diameter of the cylinder.

### Growth rate measurement

Growth rates of AB958 and MG1655 were measured in shaken cultures of 200μl liquid LB medium at 300C in 96 well microtiter plates. 16 wells per strain were inoculated from single colonies and grown to stationary phase. Cultures were diluted and transferred to fresh LB medium. For each culture, three different dilution factors were used (200, 400 and 800), resulting in a total of 48 cultures per strain. The distribution of the two strains on the 96 well plate was balanced to control for possible position effects; the two strains were arranged in a checkerboard-pattern. After dilution cultures were grown in a microplate spectrophotometer (SpectraMax 344PC from Molecular Devices), and the optical density (OD600) was determined every two minutes. The population doubling time during exponential growth was calculated based on linear regression of the log-transformed optical densities on time; linear regression was performed on OD values in the range between 0.0625 and 0.125. Only five out of 96 growth curves had a Pearson product moment correlation coefficient R2 of less than 0.99, and these five growth curves were excluded from the analysis.

### Statistical analysis

Statistical analyses were performed using the software SPSS and R. For analyzing the increase in mortality with age (Fig 1D) by means of logistic regression we had to treat each cell division of a given cell as an independent data point.

### Electron microscopy

Cells were grown in LB medium to an OD578 of 1, pelleted in a microcentrifuge tube and fixed in 4% glutaraldehyde in PBS for 2h at 4°C. After washing with PBS containing 0,25M Glucose, cells were treated with 1% OsO4 for 2,5 h at 4°C and washed with distilled H2O. Samples were dehydrated with an ethanol step gradient (50%, 70%, 90%, 100%, 10 min each) and a final acetone step (100%, 10 min). Dehydrated samples were treated for 2h with 50% “epon” [48] in acetone and pure epon for 4h. Polymerization was carried out at 60°C for 24h. 60–70 nm thin cuts were made on a Ultracut E (Reichert-Jung) and stained with 6% UrAc (1 h) and PbAc (2 min) according to Millonig [49]. Pictures were recorded on a Philips Morgagni 268D electron microscope at 80 kV.

## Supporting Information

S1 Fig Detailed history of 22 *csrA* cells. We plot cell length versus time for 22 individual mother cells over their entire lifetime. From these plots, it is possible to estimate division times, cell lengths, and growth rates.
(PDF)

S2 Fig Cell size after division is significantly smaller for last divisions in a *csrA* background. We measured cell length of the focal *csrA* cells right after division, for all pole ages at which cells divided, and analyzed whether it was the last division or not. Cell size is significantly smaller if it is the last division of a cell (blue bars) than any other division (orange bars, logistic regression/ANOVA, p = 8.9x10^−7^, N = 72). We have also analyzed this effect for two more experiments for which we do not show the plots, and the effect is also significant (p = 2.1x10^−3^, 7.9x10^−4^, N = 37, 23).
(PDF)

S3 Fig Times to last divisions of mothers and first divisions of daughters are indistinguishable in *csrA* mutant cells. We compared interdivision intervals of the first divisions of 38 focal *csrA* mutant cells, and of the first division of every focal cell’s last daughter cell. The interdivision intervals are not significantly different (N = 38, Anova, p = 0.37).
(PDF)

S4 Fig Growth curves of wild type *E*. *coli* cells and *csrA* cells. The plot depicts growth curves for 16 cultures of wild type *E*. *coli* (MG1655; purple) and 16 cultures of *csrA* (orange). Between the lag phase and the stationary phase the growth curves are close to exponential, manifesting as approximately linear growth curves on this plot with a logarithmic y-axis. Growth rates (reported in the main text) were determined by linear regression between OD600 = 0.0625 and OD600 = 0.125. The cultures that were initiated by diluting 400 times are shown (see Methods).
(PDF)

S5 Fig GlgA-GFP accumulates at old poles in *csrA* cells. The sums of all pixel intensities along the short axis of the rectangles enclosing cells were calculated for every pixel along the long axis for *csrA* cells carrying the plasmid encoding GlA-GFP. Relative intensities are plotted for the four quarters of the cell (from old to new pole, blue, green, orange, and black, see inserts) along the normalized lifetime of the cell. These intensities increase first in the quarter of the cell containing the old pole (blue), and subsequently in the neighboring quarters (first, green, then orange, then blue), until the whole cell is filled. Solid lines are the median relative fluorescence, shaded areas span the 25 to 75 percentiles. N = 78.
(PDF)

S6 Fig GlgA-GFP concentration and size after division are predictive of last divisions in a *csrA* background. We plotted GlgA-GFP concentration and cell length after division for all observed cells after every division. Red points denote last divisions, black points denote all other divisions. GlgA-GFP concentration is significantly higher (logistic regression/ANOVA, p<2.2x10^−16^), and cell size at birth is significantly smaller (logistic regression/ANOVA, p = 5.9x10^−16^) if it is a cell’s last division. N = 78.
(PDF)

S7 Fig GlgA-GFP concentration and cell length at birth are predictive of a cell’s total replicative potential. (A) GlgA-GFP concentration of emerging new-pole cells shows significant negative correlation with their replicative potential (Spearman’s rho = −0.29, p = 0.0105, N = 78). (B) Cell length of emerging new-pole cells shows significant positive correlation with their replicative potential (Spearman’s rho = 0.30, p = 0.0083). We note that the analyses in (A) and (B) are not independent of each other, since the cell length is used in calculating the GlgA-GFP concentration (see Methods).
(PDF)

S8 Fig GFP expressed under control of the promoter of the ribosomal protein RpsM shows no evidence for polar localization. A growing microcolony of *csrA* mutant cells harboring a plasmid encoding GFP controlled by the promoter of *rpsM* was observed. The GFP signal was distributed homogeneously in the cells’ cytoplasm, although the GFP signal appeared to be weaker at the poles of some cells. Red and blue arrows on the still images indicate the two poles of the cell that founded the microcolony, and the paths of the poles are indicated on the lineage tree. Size bar is 5μm.
(PDF)

S9 Fig A staining experiment supports loss of DNA from old pole cells in the *csrA* mutant. We used time-lapse microscopy to observe a microcolony of *csrA* mutant cells growing on agar containing Hoechst DNA stain. The right panel shows individual frames from the time-lapse experiment, with red and blue arrows indicating the poles of the cell that founded the microcolony, and red and blue lines on the tree indicate the paths of these poles. The grayscale squares at the tip of the tree indicate fluorescence intensity of the Hoechst DNA stain at this time point. Size bar is 5μm.
(PDF)

S1 Movie *csrA* mutant cells are grown in a microfluidic device and observed microscopically. Time between frames is 5 minutes; the timer in the top left corner indicates hh:mm:ss. The movie shows how cells with old cell poles stop growing and dividing, and how the new-pole progeny produced by these cells are rejuvenated and only suffer from the growth and division-terminating phenotype once their own cell pole is old.
(AVI)

S2 Movie *csrA* mutant cells are grown on an agar pad and form a microcolony under the microscope. Movie of a growing microcolony of the *csrA* mutant. Time between frames is 5 minutes; the timer indicates hh:mm:ss. The red dot marks the cell pole of one individual (‘Individual A’) throughout the movie. This individual is born at time 01:54:00, when the marked pole becomes the older cell pole (before birth, the cell pole is marked by an empty red dot). At the time of birth, the chronological age of the individual is 0:00:00 hours, and its replicative age is 0 divisions. Individual A divides three times in the course of 1:46 hours and then stops. At the last division, it produces a rejuvenated progeny, individual B (marked by an empty blue dot before birth, and by a full blue dot afterwards). Individual B is born at time 03:40:00 and divides four times before the movie stops.
(MOV)

S3 Movie *csrA* mutant cells expressing GlgA-GFP are grown in a microfluidic device and observed microscopically. GlgA-GFP colocalizes with glycogen. Time between frames is 10min, the timer in the top left corner indicates hh:mm:ss.
(AVI)

S4 Movie wild type cells expressing GlgA-GFP are grown in a microfluidic device and observed microscopically. GlgA-GFP colocalizes with glycogen. Time between frames is 10min, the timer in the top left corner indicates hh:mm:ss.
(AVI)

S5 Movie *csrA* mutant cells expressing Hns-GFP are grown in a microfluidic device and observed microscopically. Hns-GFP colocalizes with DNA. Time between frames is 10min, the timer in the top left corner indicates hh:mm:ss.
(AVI)

S1 Text Quantification of the growth rate reduction as a consequence of age-specific mortality. (PDF)
