# Supplementary figures and images for: Genetic Manipulation of Glycogen Allocation Affects Replicative Lifespan in E. coli
Source: PLoS Genet. 2016 Apr 19;12(4):e1005974. doi: 10.1371/journal.pgen.1005974 (PMC4836754; doi:10.1371/journal.pgen.1005974)

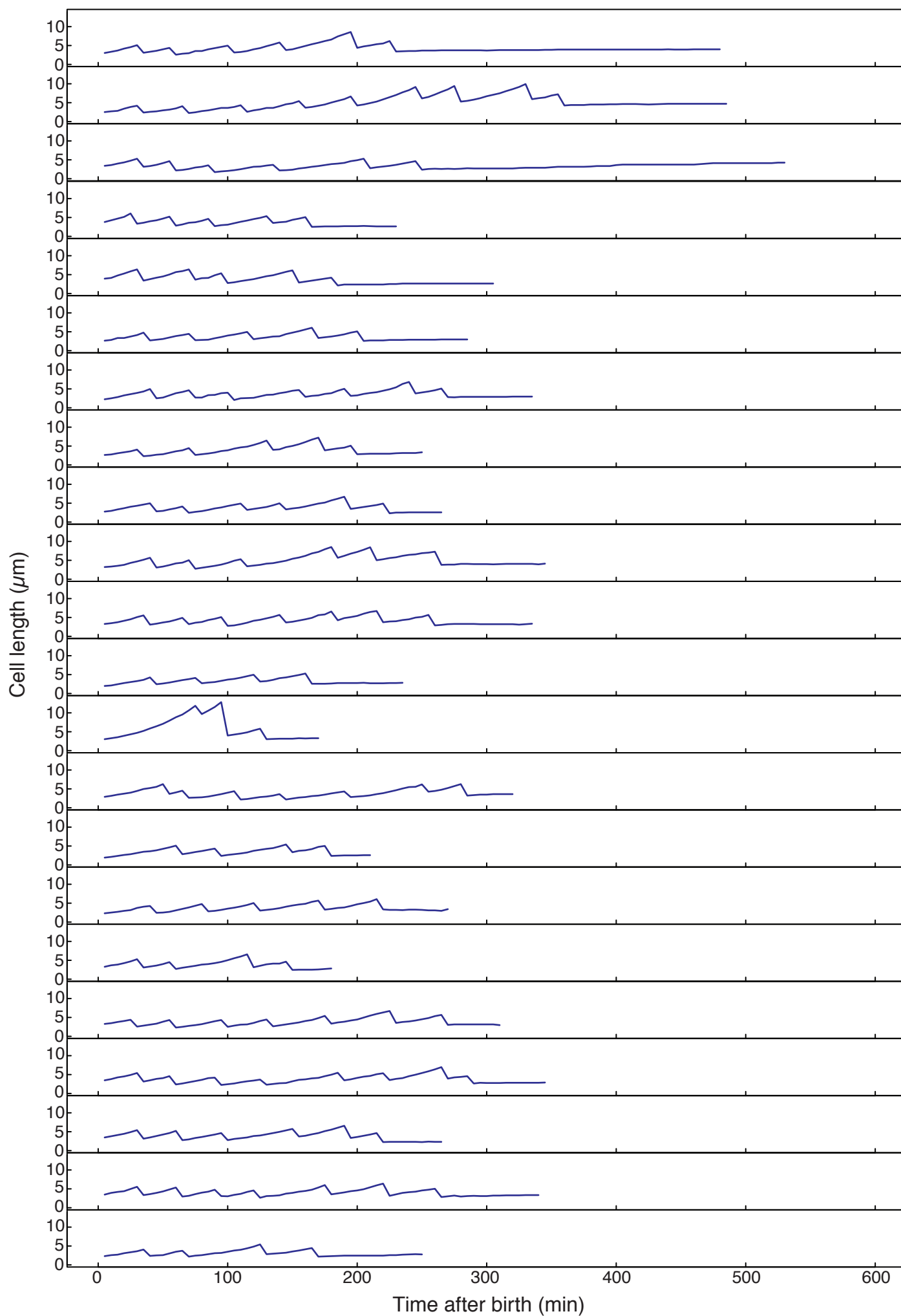

**Fig. S1**

Supplement: S1 Fig — We plot cell length versus time for 22 individual mother cells over their entire lifetime. From these plots, it is possible to estimate division times, cell lengths, and growth rates. (PDF) [file pgen.1005974.s001.pdf]

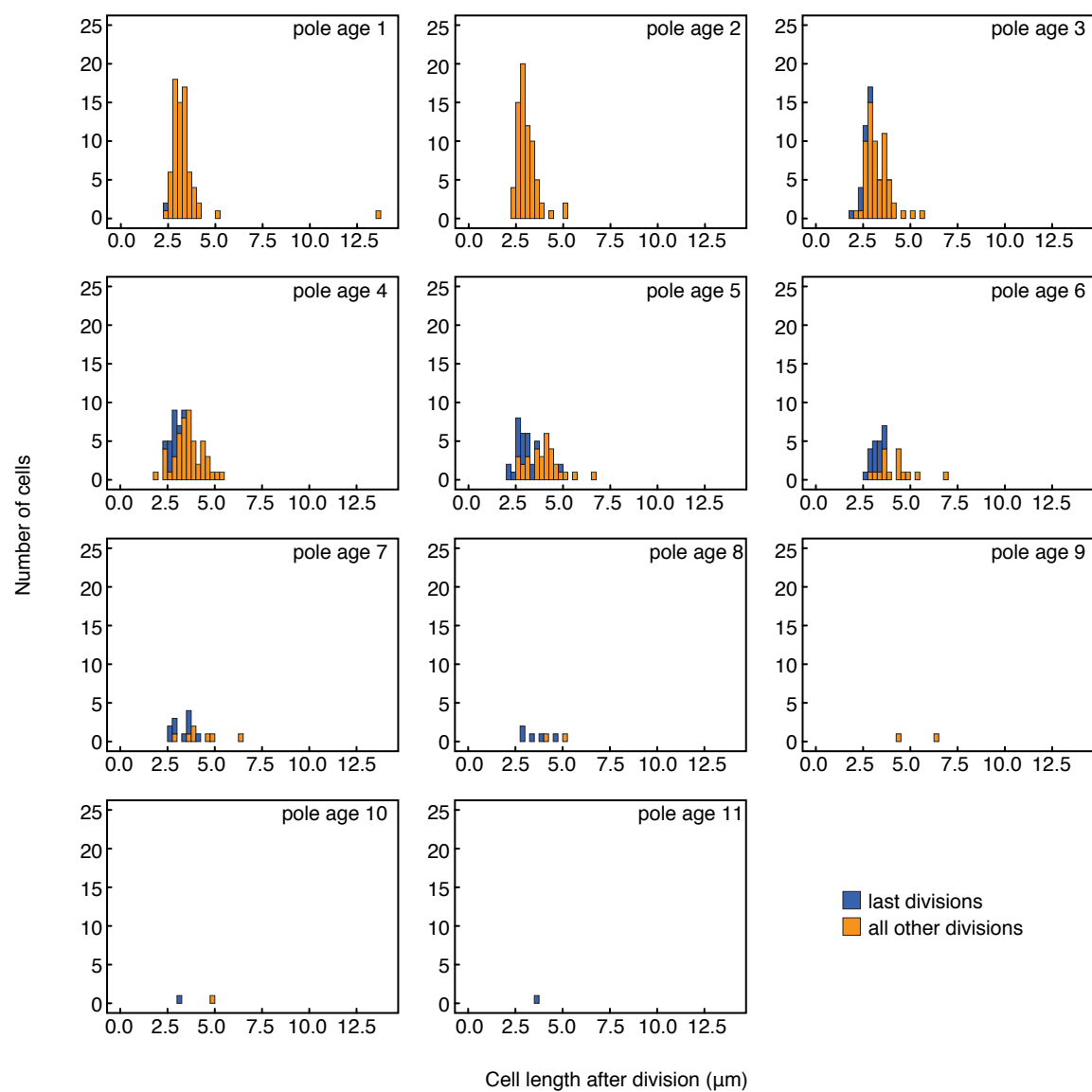

**Fig. S2**

Supplement: S2 Fig — We measured cell length of the focal csrA cells right after division, for all pole ages at which cells divided, and analyzed whether it was the last division or not. Cell size is significantly smaller if it is the last division of a cell (blue bars) than any other division (orange bars, logistic regression/ANOVA, p = 8.9x10^−7^, N = 72). We have also analyzed this effect for two more experiments for which we do not show the plots, and the effect is also significant (p = 2.1x10^−3^, 7.9x10^−4^, N = 37, 23). (PDF) [file pgen.1005974.s002.pdf]

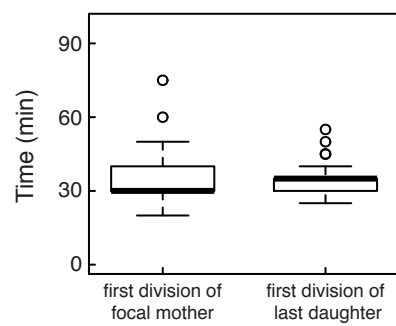

**Fig. S3**

Supplement: S3 Fig — We compared interdivision intervals of the first divisions of 38 focal csrA mutant cells, and of the first division of every focal cell’s last daughter cell. The interdivision intervals are not significantly different (N = 38, Anova, p = 0.37). (PDF) [file pgen.1005974.s003.pdf]

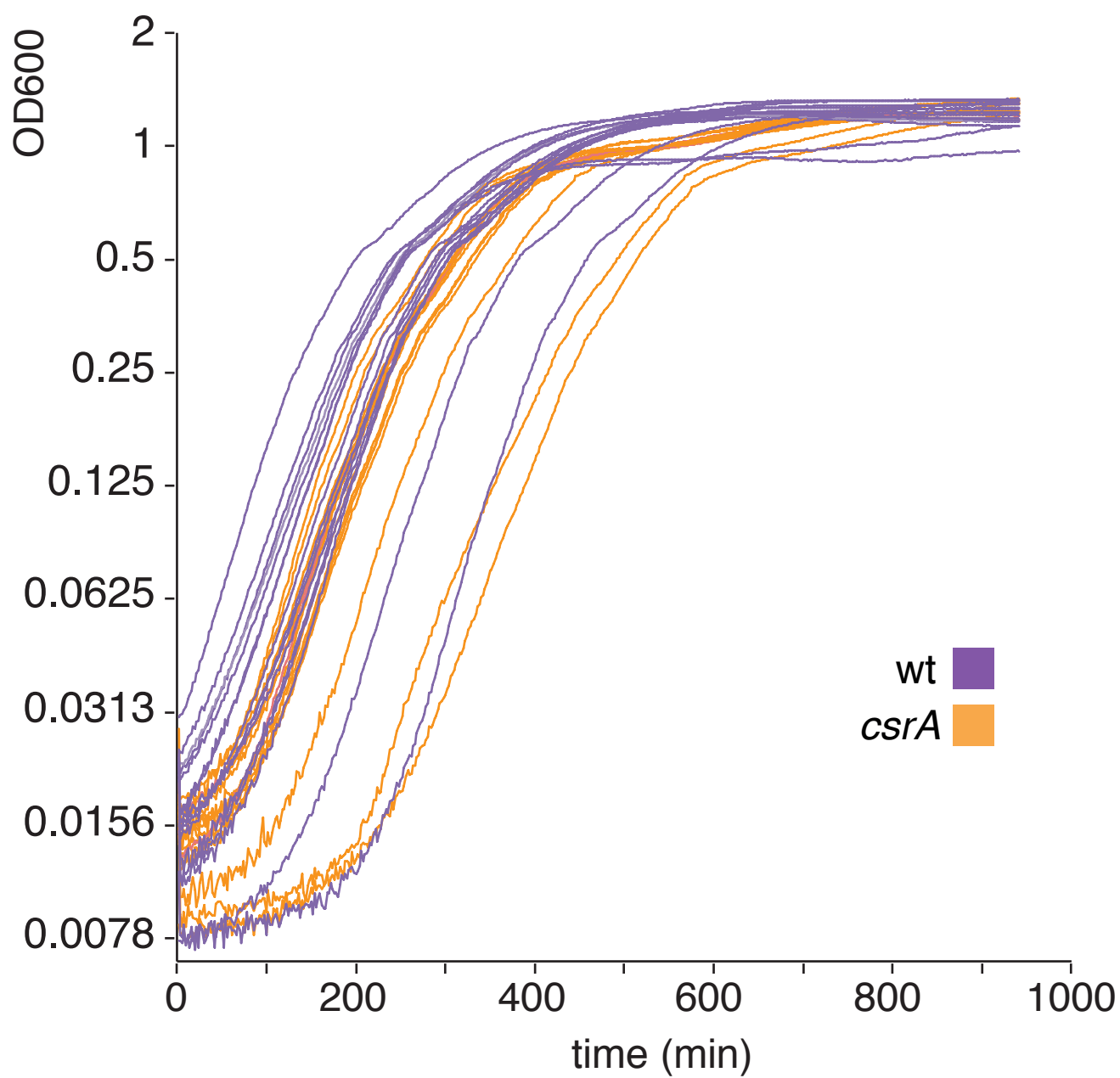

**Fig. S4**

Supplement: S4 Fig — The plot depicts growth curves for 16 cultures of wild type E. coli (MG1655; purple) and 16 cultures of csrA (orange). Between the lag phase and the stationary phase the growth curves are close to exponential, manifesting as approximately linear growth curves on this plot with a logarithmic y-axis. Growth rates (reported in the main text) were determined by linear regression between OD600 = 0.0625 and OD600 = 0.125. The cultures that were initiated by diluting 400 times are shown (see Methods). (PDF) [file pgen.1005974.s004.pdf]

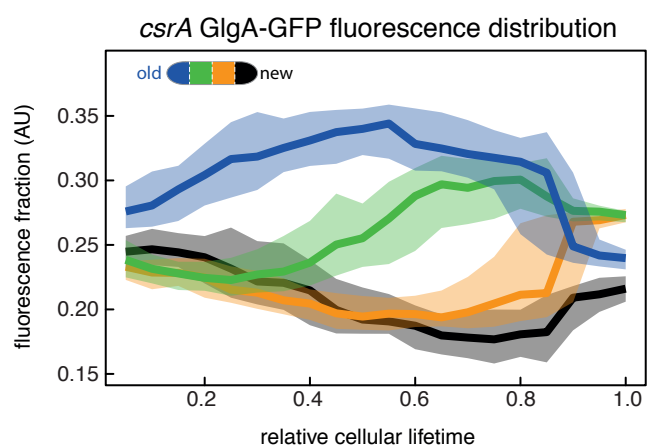

**Fig. S5**

Supplement: S5 Fig — The sums of all pixel intensities along the short axis of the rectangles enclosing cells were calculated for every pixel along the long axis for csrA cells carrying the plasmid encoding GlA-GFP. Relative intensities are plotted for the four quarters of the cell (from old to new pole, blue, green, orange, and black, see inserts) along the normalized lifetime of the cell. These intensities increase first in the quarter of the cell containing the old pole (blue), and subsequently in the neighboring quarters (first, green, then orange, then blue), until the whole cell is filled. Solid lines are the median relative fluorescence, shaded areas span the 25 to 75 percentiles. N = 78. (PDF) [file pgen.1005974.s005.pdf]

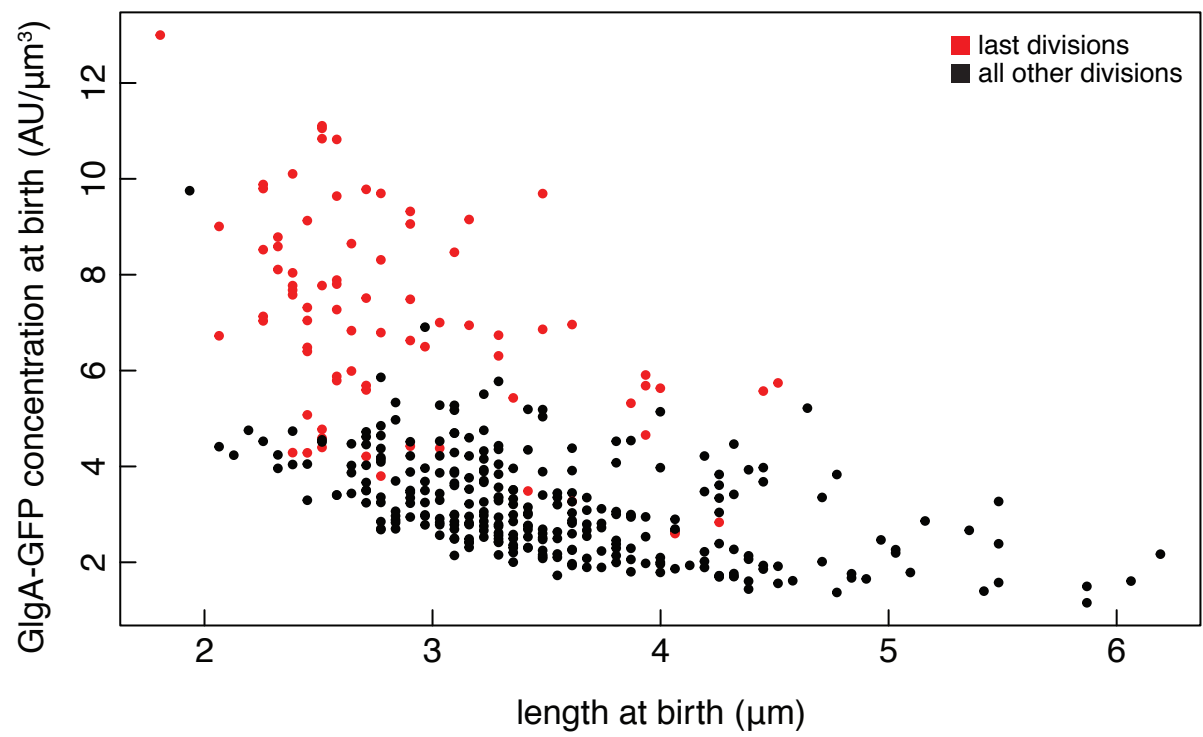

**Fig. S6**

Supplement: S6 Fig — We plotted GlgA-GFP concentration and cell length after division for all observed cells after every division. Red points denote last divisions, black points denote all other divisions. GlgA-GFP concentration is significantly higher (logistic regression/ANOVA, p<2.2x10^−16^), and cell size at birth is significantly smaller (logistic regression/ANOVA, p = 5.9x10^−16^) if it is a cell’s last division. N = 78. (PDF) [file pgen.1005974.s006.pdf]

**A**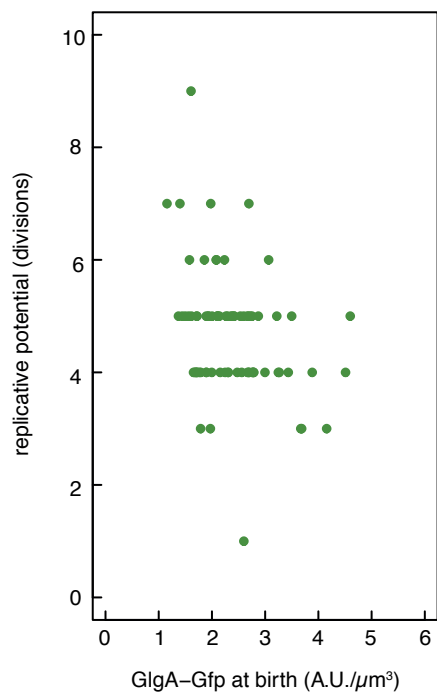**B**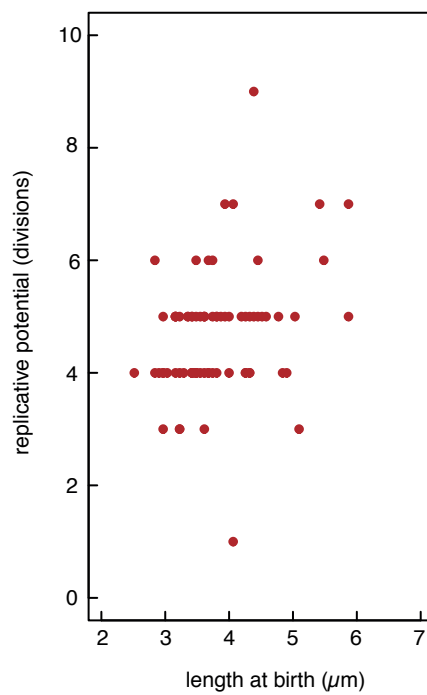**Fig. S7**

Supplement: S7 Fig — (A) GlgA-GFP concentration of emerging new-pole cells shows significant negative correlation with their replicative potential (Spearman’s rho = −0.29, p = 0.0105, N = 78). (B) Cell length of emerging new-pole cells shows significant positive correlation with their replicative potential (Spearman’s rho = 0.30, p = 0.0083). We note that the analyses in (A) and (B) are not independent of each other, since the cell length is used in calculating the GlgA-GFP concentration (see Methods). (PDF) [file pgen.1005974.s007.pdf]

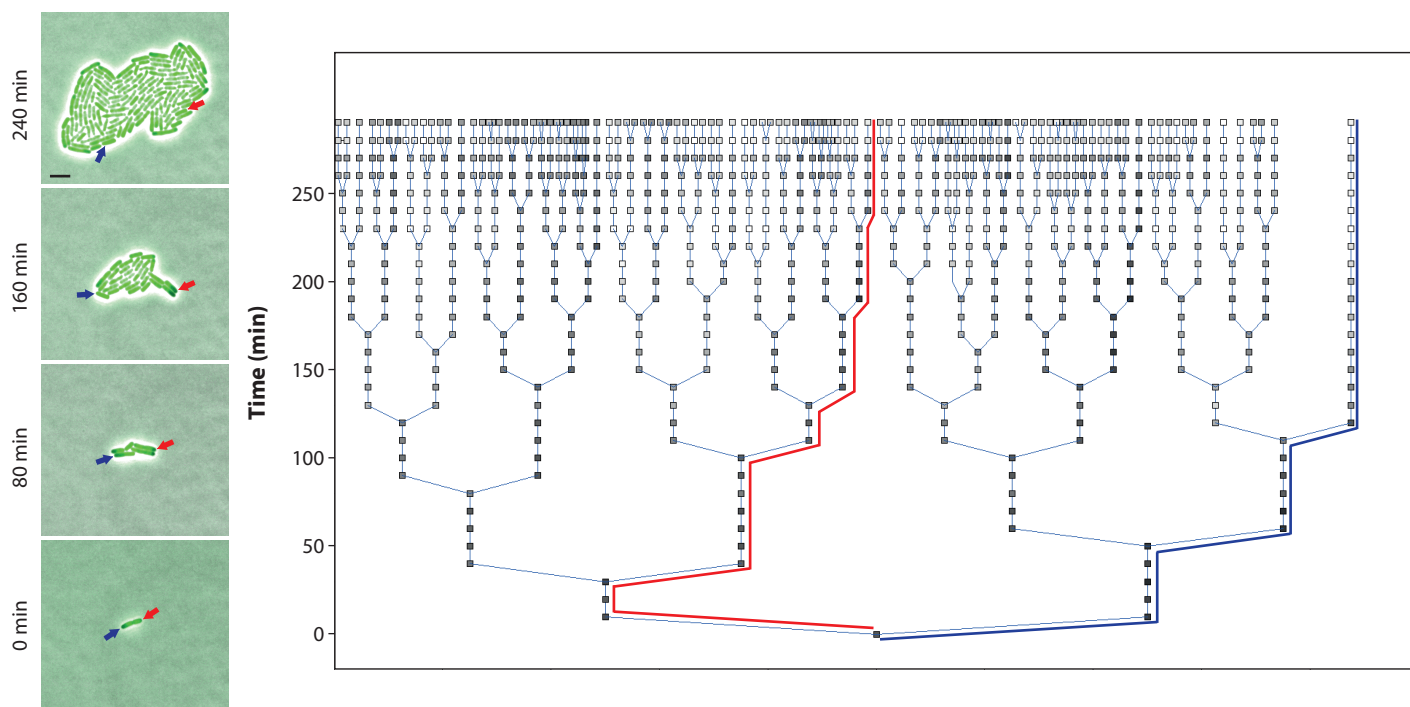

**Fig. S8**

Supplement: S8 Fig — A growing microcolony of csrA mutant cells harboring a plasmid encoding GFP controlled by the promoter of rpsM was observed. The GFP signal was distributed homogeneously in the cells’ cytoplasm, although the GFP signal appeared to be weaker at the poles of some cells. Red and blue arrows on the still images indicate the two poles of the cell that founded the microcolony, and the paths of the poles are indicated on the lineage tree. Size bar is 5μm. (PDF) [file pgen.1005974.s008.pdf]

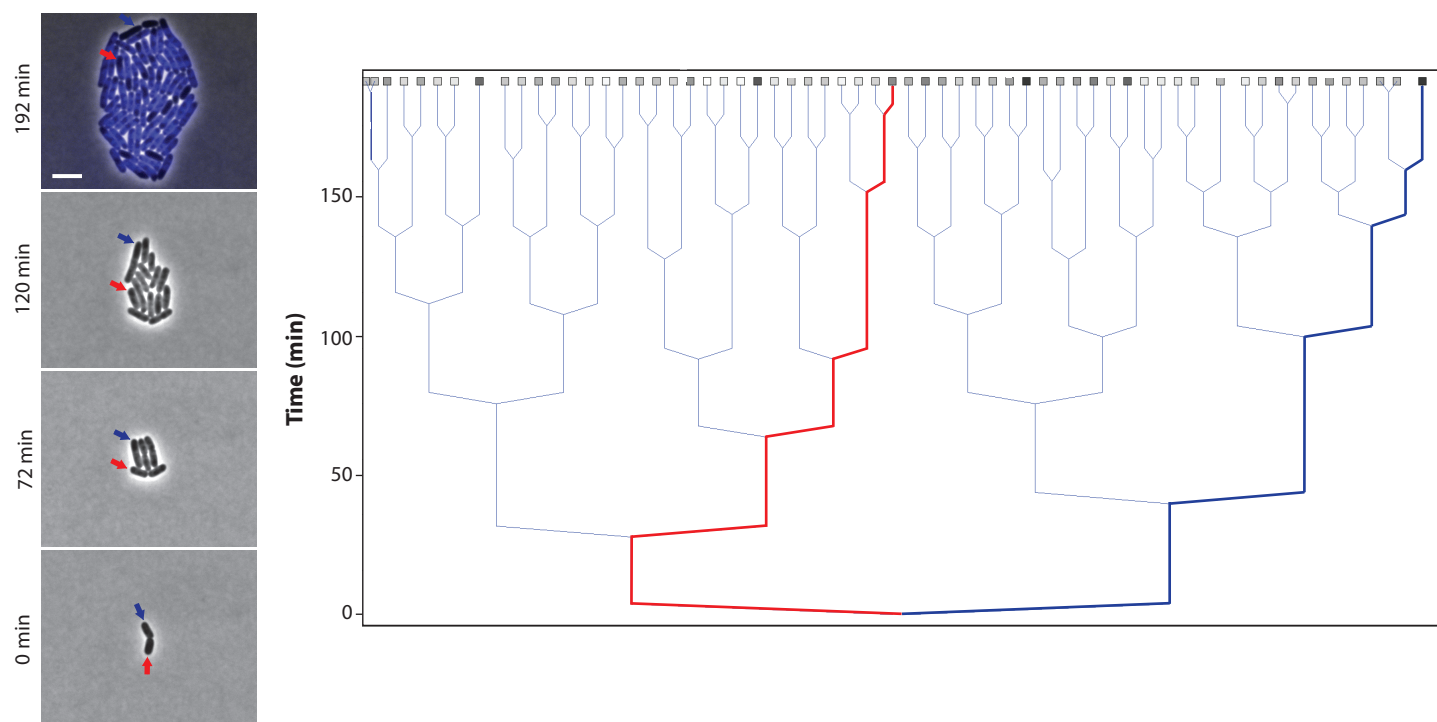

**Fig. S9**

Supplement: S9 Fig — We used time-lapse microscopy to observe a microcolony of csrA mutant cells growing on agar containing Hoechst DNA stain. The right panel shows individual frames from the time-lapse experiment, with red and blue arrows indicating the poles of the cell that founded the microcolony, and red and blue lines on the tree indicate the paths of these poles. The grayscale squares at the tip of the tree indicate fluorescence intensity of the Hoechst DNA stain at this time point. Size bar is 5μm. (PDF) [file pgen.1005974.s009.pdf]
