# Supplementary material for: Genetic Manipulation of Glycogen Allocation Affects Replicative Lifespan in E. coli
Source: PLoS Genet. 2016 Apr 19;12(4):e1005974. doi: 10.1371/journal.pgen.1005974 (PMC4836754; doi:10.1371/journal.pgen.1005974)
Supplement: S1 Text — (PDF) [file pgen.1005974.s015.pdf]

Supplementary Text 1: Quantification of the growth rate reduction as a consequence of age-specific mortality

We used the Euler-Lotka equation (Charlesworth, 1994) to determine how a given pattern of age-specific mortality (as observed in the *csrA* mutant) influences the population growth rate. For a bacterium that reproduces by producing a young-pole cell at regular intervals, and that has a given pattern of intrinsic mortality that changes with pole age, the long-term population growth rate *r* of a strain is approximately defined by $1 = \sum_{n=0 \text{ to } m} l_n{*}e^{-r(bn)}$, where *n* is a counter for divisions, *m* is the maximal number of divisions that a cell can possibly achieve, l_n is the probability to survive to an age of n, and *b* is the time interval between consecutive divisions. This is a simplified version of the Euler-Lotka equation for discrete time, where age-specific output is equal to one for all ages where a cell divides, and zero otherwise. The equation is different from what we used in an earlier publication (Ackermann, Schauerte, Stearns, & Jenal, 2007) for two reasons: first, here we assumed that the interval between all cell divisions is constant, while in (Ackermann et al., 2007) we modeled the bacterium *Caulobacter crescentus* whose interdivision interval for new-pole cells is higher that the other interdivision intervals. Second, here we were interested in strains with age-specific intrinsic mortality, while in (Ackermann et al., 2007) we modeled bacteria that had no intrinsic mortality.
Evaluating the equation numerically for different age-specific mortality patterns l_n reveals how *r* depends on l_n, and thus the growth rate cost of a mutation that leads to a given pattern of intrinsic age-specific mortality (the growth rate cost was calculated for *m* = 30. Using larger *m* did not have an effect that was large enough to be detectable given the numerical precision we were working with).
We used the age-specific survival data shown in Table S1 for *csrA* and wild type *E. coli*.

n (age in divisions)	l_n *csrA*	l_x wild type
1	0.982	0.991
2	0.982	0.980
3	0.908	0.980
4	0.700	0.980
5	0.424	0.980
6	0.236	0.975
7	0.096	0.971
8	0.035	0.971
9	0.009	0.962
10	0.009	0.962
11	0.000	0.962
≧12	0.000	0.962

Table S1: l_n, the probability to survive to divisional age n, for the *csrA* strain and for wild type *E. coli*. This data is based on the experiments shown in Fig. 1C.

For both strains, we assumed that the interdivision interval was constant at 40 minutes. This assumption is consistent with single-cell observations of interdivision intervals from microfluidic experiments (the same experiment for which the data is shown in Fig. 1C).

Solving this equation numerically, we found that the population growth rate of *csrA* is predicted to be only about **6.45%** lower than the population growth rate of wild type, as a consequence of the different survival pattern l_n.

We extended this analysis to ask the following question: by how many divisions would the negative effect need to be delayed so that the reduction in the population growth rate was 10^{-5} or less? (as discussed in the main text, mutations with a deleterious effect that is so small cannot be efficiently removed by natural selection from bacterial populations). For this extension we assumed that the base-line mortality rate (i.e., the mortality rate before the age-dependent increase in the *csrA* mutant) for both the wt and the *csrA* mutant was zero, which is very close to the value for the wt, and that mortality of the *csrA* mutant would increase as shown in in Table S1 after a fixed delay (in number of divisions). We constructed modified tables of l_x, the probability to survive to age x, and solved the Euler Lotka equation (above) nummerically for these l_x tables. Doing so we found that delaying the deleterious effect by 13 divisions leads to a predicted reduction in the population growth rate *r* of $7*10^{-6}$, i.e., less than 10^{-5}. This means that if this deleterious effect (the increase in mortality rate) of the *csrA* mutation would only manifest at a replicative age of 17 or 18 divisions (instead of 4 to 5 divisions as reported in Fig. 1C in the main text) then the resulting reduction in population growth rate *r* would be so small that the *csrA* mutation would not be efficiently removed from bacterial populations.

References:

Ackermann, M., Schauerte, A., Stearns, S. C., & Jenal, U. (2007). Experimental evolution of aging in a bacterium. *BMC Evolutionary Biology*, *7*, 126.

Charlesworth, B. (1994). *Evolution in age-structured populations*. Cambridge: Cambridge University Press.
